# Diagnostic stability and long-term outcomes of acute and transient psychotic disorders: a systematic review and meta-analysis

**DOI:** 10.3389/fpsyt.2026.1839599

**Published:** 2026-07-10

**Authors:** Qian He, Yi-chao Wang

**Affiliations:** Affiliated Mental Health Center & Hangzhou Seventh People’s Hospital, Zhejiang University School of Medicine, Hangzhou, Zhejiang, China

**Keywords:** acute and transient psychotic disorders, diagnostic trajectory, diagnostic transition, longitudinal outcomes, meta-analysis, relapse, schizophrenia spectrum

## Abstract

**Background:**

To examine the diagnostic stability and long-term outcomes of acute and transient psychotic disorders (ATPD), with particular attention to subsequent diagnostic transitions and relapse over time.

**Methods:**

A systematic search of PubMed, Embase, Web of Science, the Cochrane Library, as well as Chinese databases including CNKI, Wan fang, and VIP, was conducted from database inception to March 1, 2026. Observational studies reporting follow-up outcomes in patients initially diagnosed with ATPD were eligible. Data extraction and quality assessment were performed independently by two reviewers. Pooled proportions were estimated using random-effects models, and statistical analyses were conducted using R software (version 4.5.1).

**Results:**

A total of 14 studies involving 12,920 participants were included. The pooled proportion of diagnostic stability was 0.41 (95% CI: 0.34–0.51), while 0.50 (95% CI: 0.36–0.63) of patients experienced any diagnostic change. Transition to schizophrenia spectrum disorders occurred in 0.39 (95% CI: 0.29–0.50) of cases, whereas transition to mood disorders was less frequent (0.19, 95% CI: 0.12–0.30). The pooled relapse rate was 0.43 (95% CI: 0.29–0.58).

**Conclusions:**

ATPD appears to show limited diagnostic stability, with a considerable proportion of patients experiencing diagnostic change over time. Transitions to schizophrenia spectrum disorders are relatively common, whereas affective outcomes are less frequent. However, substantial heterogeneity across studies limits the certainty of these estimates. These findings underscore the need for cautious interpretation of early diagnoses and highlight the importance of longitudinal follow-up. Further prospective studies with standardized methodologies are warranted.

**Systematic review registration:**

https://www.crd.york.ac.uk/PROSPERO/view/CRD420261347946, identifier CRD420261347946.

## Introduction

Acute and transient psychotic disorder (ATPD) is an ICD-defined category of short-lived psychotic disorders characterized by the abrupt onset of psychotic symptoms, including delusions, hallucinations, disorganized speech, perceptual disturbance, or markedly disorganized behavior (1). Symptoms usually develop rapidly, often within days to two weeks, and are expected to remit within a relatively short period. In ICD-10, ATPD includes several subtypes according to the presence of polymorphic symptoms and schizophrenia-like features, whereas ICD-11 retains the core concept of an acute, brief, and remitting psychotic disorder (2). Brief psychotic disorder is the closest DSM-based counterpart, defined by the presence of one or more core psychotic symptoms lasting at least one day but less than one month, followed by return to the premorbid level of functioning (3, 4). Although these constructs overlap clinically, ATPD and brief psychotic disorder are not fully interchangeable because ICD and DSM systems differ in symptom requirements, duration thresholds, exclusion rules, and the extent to which polymorphic symptom changes or stress-related onset are emphasized (5).

The diagnostic boundaries and prognostic meaning of ATPD remain clinically important but uncertain. Traditionally, ATPD has been regarded as a relatively favorable and self-limited psychotic condition compared with schizophrenia spectrum disorders (6, 7). However, clinical follow-up studies have shown that not all patients retain the initial diagnosis over time. Some individuals remain diagnostically stable after remission, whereas others experience relapse or later receive a diagnosis of schizophrenia spectrum disorders, affective psychoses, or other psychiatric conditions (8). Therefore, diagnostic stability is not merely a methodological indicator, but a clinically meaningful outcome reflecting whether the initial ATPD diagnosis represents a transient episode, a provisional early-stage diagnosis, or the first manifestation of a more persistent psychiatric disorder (9).

Clarifying the long-term course of ATPD is relevant to both clinical management and early psychosis research. For clinicians, an accurate understanding of diagnostic stability and transition risk may inform follow-up intensity, treatment planning, psychoeducation, and patient counseling (10). Overestimating the risk of chronic psychotic illness may lead to unnecessary long-term treatment and stigma, whereas underestimating the possibility of diagnostic transition may delay monitoring and timely intervention. For researchers, ATPD provides an important model for examining how brief and acute psychotic presentations evolve across diagnostic systems and clinical settings (11).

Existing longitudinal studies (12, 13) have reported heterogeneous estimates of diagnostic stability, relapse, and transition to schizophrenia spectrum or mood disorders. These discrepancies may reflect differences in diagnostic criteria, study design, sample source, follow-up duration, treatment exposure, and outcome definitions. Individual studies are often limited by sample size, setting-specific case ascertainment, or inconsistent operationalization of diagnostic transition and relapse. As a result, the overall long-term trajectory of ATPD remains insufficiently defined, and pooled evidence is needed to summarize available data while explicitly accounting for between-study heterogeneity (14).

Therefore, the study aimed to synthesize longitudinal evidence on diagnostic stability and long-term outcomes among individuals initially diagnosed with ATPD or closely related brief psychotic disorder constructs. Specifically, we estimated the pooled proportions of diagnostic stability, any diagnostic change, transition to schizophrenia spectrum disorders, transition to mood disorders, and relapse. We also examined potential sources of heterogeneity where data permitted, with the aim of providing a clearer and more cautious summary of the prognostic course of ATPD.

## Methods

This systematic review and meta-analysis were conducted in accordance with the Preferred Reporting Items for Systematic Reviews and Meta-Analyses (PRISMA 2020) guidelines (15). This study was registered with Prospero, with the registration number: CRD420261347946.

### Search strategy

A comprehensive literature search was performed in PubMed, Embase, Web of Science, and the Cochrane Library, as well as Chinese databases including CNKI, Wan fang, and VIP, from database inception to March 1, 2026. No language restrictions were applied. The search strategy combined controlled vocabulary terms and free-text keywords related to acute and transient psychotic disorders and longitudinal outcomes. The search strategy combined controlled vocabulary terms and free-text keywords for ATPD, brief psychotic disorder, and longitudinal diagnostic outcomes. To maximize sensitivity, broader early-psychosis terms were also included because some first-episode psychosis cohorts may report ATPD- or brief psychotic disorder-specific subgroups. Search terms therefore included “acute and transient psychotic disorder,” “acute transient psychotic disorder,” “ATPD,” “brief psychotic disorder,” “brief reactive psychosis,” “reactive psychosis,” “short-lived psychosis,” “first episode psychosis,” “first-episode psychosis,” “first admission psychosis,” “first-admission psychosis,” and “early psychosis,” combined with terms such as “diagnostic stability,” “diagnostic change,” “diagnostic transition,” “course,” “outcome,” “prognosis,” “follow-up,” “relapse,” and “conversion.” The full search strategy for each database is provided in Supplementary Material **Supplementary Table 1**. In addition, the reference lists of included studies and relevant reviews were manually screened to identify further eligible studies.

### Eligibility criteria

#### Inclusion criteria

Studies were eligible if they included individuals with an initial diagnosis of acute and transient psychotic disorder (ATPD) or a closely corresponding short-lived psychotic disorder construct, such as brief psychotic disorder, according to ICD, DSM, or clearly defined comparable diagnostic criteria. The index diagnosis had to be ATPD or its equivalent at baseline or first presentation. Eligible studies were required to use a longitudinal observational design and to report at least one follow-up outcome, including diagnostic stability, diagnostic transition, transition to schizophrenia spectrum disorders, transition to mood disorders, relapse, or any diagnostic change.

Studies were excluded if the baseline population consisted primarily of schizophrenia, schizophreniform disorder, schizoaffective disorder, affective psychosis, substance-induced psychosis, organic psychosis, or other acute psychotic disorders without ATPD-specific extractable data. Studies including mixed psychotic disorder samples were retained only when ATPD-specific baseline data and follow-up outcomes could be extracted separately. When schizophreniform disorder, schizophrenia, or other psychotic disorders emerged during follow-up among patients initially diagnosed with ATPD, these diagnoses were treated as outcome events indicating diagnostic transition rather than as exclusion criteria.

#### Data extraction

Data extraction was conducted independently by two reviewers using a standardized data collection form. The following variables were extracted from each included study: study characteristics (first author, publication year, country, and study design), data source, sample size, and participant characteristics (mean or median age and proportion of female participants). Clinical and methodological details were also recorded, including diagnostic criteria (ICD-10, ICD-11, or DSM), study setting (inpatient, outpatient, or mixed), whether the sample included first-episode cases only, and duration of follow-up. In addition, outcome definitions were extracted, including criteria used to define diagnostic stability, diagnostic transition, and relapse. Any discrepancies were resolved through discussion or consultation with a third reviewer (Fu-gang Luo), When information was unclear or incomplete, extraction was based on the published article and **Supplementary Materials**. No unpublished author-provided data were included in the quantitative synthesis. Discrepancies in data extraction were resolved through discussion and consensus. When necessary, methodological advice was sought from an independent consultant.

### Quality assessment

The methodological quality of included studies was assessed using the Newcastle–Ottawa Scale (NOS) (16) for observational studies. This tool evaluates studies based on selection, comparability, and outcome domains. Studies were categorized as low, moderate, or high quality according to standard thresholds. Quality assessment was performed independently by two reviewers, with discrepancies resolved by consensus.

### Statistical analysis

Meta-analyses were performed using R software (version 4.5.1). Pooled proportions and corresponding 95% confidence intervals (CIs) were calculated using random-effects models (DerSimonian–Laird method), given the anticipated clinical and methodological heterogeneity across studies. Where appropriate, transformations were applied to stabilize variances prior to pooling.

Between-study heterogeneity was assessed using the I² statistic, with values of 25%, 50%, and 75% representing low, moderate, and high heterogeneity, respectively. Sensitivity analyses were conducted using a leave-one-out approach, in which each study was sequentially excluded to evaluate the robustness of the pooled estimates.

Publication bias was assessed by visual inspection of funnel plots and further examined using Egger’s regression test. Meta-regression analyses were performed to explore potential sources of between-study heterogeneity when sufficient data were available. The final examined covariates included first-episode-only sample, publication year, country/region, study design, and diagnostic criteria. Publication year was coded as a continuous numeric variable. First-episode-only sample, country/region, study design, and diagnostic criteria were coded as categorical factor variables. Mean age, proportion of female participants, and follow-up duration were extracted where reported; however, because these variables were not consistently available across studies and outcomes, they were not included in the final meta-regression models. Given the limited number of studies, all meta-regression analyses were performed as exploratory univariable models. Because of the limited number of studies for several outcomes, meta-regression analyses were performed as univariable models rather than multivariable models to avoid model overfitting. Meta-regression was not performed when fewer than an adequate number of studies reported the relevant covariate. The results were interpreted cautiously because study-level meta-regression is exploratory and may be underpowered when the number of included studies is small.

Given the limited number of studies available for certain outcomes, subgroup analyses were not performed to avoid unstable or potentially misleading estimates. All statistical tests were two-sided, and a P value < 0.05 was considered statistically significant.

## Results

### Study selection results

**Figure 1:**
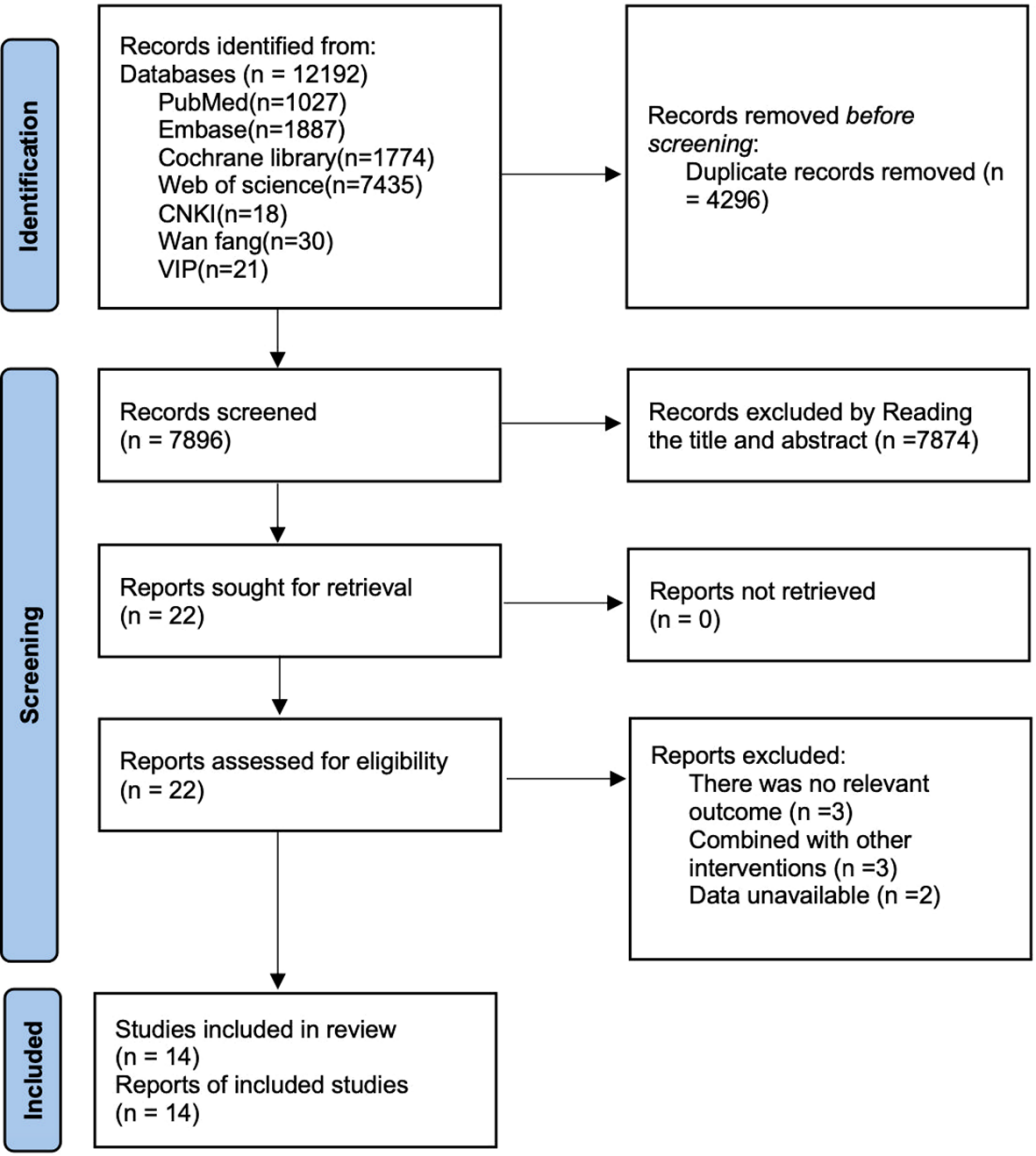
PRISMA flow diagram of the study selection process.

A total of 12,192 records were identified through database searching, including PubMed (n = 1,027), Embase (n = 1,887), the Cochrane Library (n = 1,774), Web of Science (n = 7,435), CNKI (n = 18), Wanfang (n = 30), and VIP (n = 21). After removal of 4,296 duplicates, 7,896 records remained for title and abstract screening. Of these, 7,874 records were excluded because they were unrelated to ATPD, did not report longitudinal outcomes, were not original studies, or did not include an eligible population. Twenty-two full-text articles were assessed for eligibility, and eight were excluded for the following reasons: no ATPD-specific extractable data, absence of longitudinal follow-up outcomes, insufficient numerical data for meta-analysis, or overlapping study populations. Ultimately, 14 studies (17–30) were included in the systematic review and meta-analysis. The study selection process is presented in **Figure 1**.

### Study characteristics results

The characteristics of the included studies are summarized in **Table 1**. A total of 14 studies involving 12,920 participants were included, the studies were published between 2003 and 2025 and were conducted across Europe and Asia. The mean age of participants was generally in early to mid-adulthood, although age was not consistently reported. Where available, the proportion of female participants ranged from 46.2% to 60.7%. All studies primarily used ICD-10 criteria for ATPD. Most studies focused on first-episode patients and were conducted in registry-based or hospital settings. Follow-up duration varied from 3 months to 20 years. Outcomes commonly included diagnostic stability, diagnostic transition, and relapse, although definitions differed across studies.

**Table 1:** Characteristics of the included studies.

Study	Country	Design	Data source	Sample size (N)	Age (mean ± SD/median)	Female (%)	Diagnostic criteria	Setting	First episode only	Follow-up duration	Outcome definition
Castagnini et al., 2014 (17)	Denmark	Retrospective cohort	Danish Psychiatric Central Register	5,426	15–64 years	NR	ICD-10 (F23)	National psychiatric registry (inpatient + outpatient)	Yes	Mean 9.3 years	Course, diagnostic stability, and transition of ATPD subtypes/acute polymorphic psychotic disorder
Damiani et al., 2021 (18)	UK	Retrospective EHR cohort study	South London and Maudsley NHS electronic health records	3,018	33.75 ± 14.01 years	52.70%	ICD-10 (F23)	Secondary mental health care/EHR cohort	Yes	1042 ± 1011 days (up to 8 years)	Psychotic recurrence after first ATPD diagnosis
Jäger et al., 2003 (19)	Germany	Follow-up cohort study	First-hospitalized patient sample	73	33.1 years	48%	ICD-10 (F23)	Psychiatric inpatients	Yes	3–7 years	Relapses and social adjustment/social impairment
Kar et al., 2016	India	Observational follow-up study	Tertiary care hospital sample	140	25.76 ± 9.89 years	57.90%	ICD-10 (F23)	Tertiary care hospital/outpatient follow-up	No	3 months	Diagnostic stability during the index episode
Kathfar et al., 2025 (21)	India	Retrospective record-based study	Outpatient records at tertiary care center	54	35.56 ± 17.46 years	53.70%	ICD-10 ATPD; diagnostic shifts discussed against ICD-11/DSM-5	Tertiary care center/outpatient records	Yes	6 months and 1 year	Diagnostic stability of ATPD over 1 year
López-Díaz et al., 2021 (9)	Spain	2-year cohort study	Hospital cohort	68	NR	NR	ICD-10 (F23.0–F23.9)	Clinical cohort/first-episode psychosis	Yes	2 years	Psychotic relapse and diagnostic shift
Lu et al., 2024 (22)	China	Retrospective cohort study	Hospital inpatient records and readmission records	396	31.76 ± 11.81 years	54.00%	ICD-10	Psychiatric inpatients	No	Up to 5 years	Transition from ATPD to schizophrenia
Poon & Leung, 2016	China	20-year retrospective follow-up study	Regional hospital records/Hospital Authority database	87	NR	NR	ICD-10	First-admitted psychiatric inpatients	Yes	Average 20 years	Diagnostic revision and long-term outcome
Rusaka & Rancāns, 2014 (25)	Latvia	Prospective follow-up study	Riga Centre of Psychiatry and Addiction Disorders	102	NR	60.70%	ICD-10	First-time admitted psychiatric inpatients	Yes	Mean 26.5 months	Diagnostic stability, readmission, and conversion to schizophrenia
Rutigliano et al., 2018 (26)	UK	Retrospective cohort study	South London and Maudsley NHS electronic health records	3,074	33.84 ± 13.99 years	47.0% female (53.0% male)	ICD-10 (F23)	Secondary mental health care/EHR cohort	Yes	Mean 1495 days (up to 8 years)	Transition to persistent non-organic psychotic disorders/schizophrenia-spectrum disorders
Yang et al., 2010 (30)	China	Retrospective follow-up study	First-admission inpatient cohort	112	NR	NR	ICD-10 (F23)	Psychiatric inpatients	Yes	1.0–4.5 years	Final diagnostic outcome and predictors of schizophrenia
Yu 2011 (27)	China	Follow-up study	Rehospitalization investigation + outpatient + telephone follow-up	75	NR	NR	NR	Discharged patients with ATPD	No	2–5 years	Follow-up diagnosis/diagnostic consistency
Liu, 2015 (28)	China	Retrospective analysis	Clinical records from psychiatric hospital	156	30.8 ± 10.9 years	46.20%	ICD-10 (F23)	First-admission psychiatric inpatients	Yes	NR	Prognosis/diagnostic change, mainly to schizophrenia
Wu et al., 2020 (29)	China	Retrospective study	Inpatient records + outpatient records + telephone follow-up	139	30.8 ± 11.5 years	51.80%	ICD-10	First-admission psychiatric inpatients	Yes	1–5 years	Outcome after first ATPD diagnosis/conversion to schizophrenia

### Quality assessment results

The quality assessment of the included studies is presented in **Table 2**. The NOS scores ranged from 5 to 9, indicating overall moderate to high methodological quality. A total of five studies were rated as high quality (NOS score ≥7), while the remaining studies were of moderate quality. No study was classified as low quality. Most studies performed well in the selection and outcome domains, whereas comparability scores were generally lower, reflecting limited adjustment for potential confounding factors.

**Table 2:** Quality assessment of included studies using the Newcastle–Ottawa Scale.

Study	Selection (0–4)	Comparability (0–2)	Outcome (0–3)	Total score (0–9)	Quality
Castagnini et al., 2014 (17)	4	1	3	8	High
Damiani et al., 2021 (18)	4	2	3	9	High
Jäger et al., 2003 (19)	3	1	2	6	Moderate
Kar et al., 2016	3	1	2	6	Moderate
Kathfar et al., 2025 (21)	3	1	2	6	Moderate
López-Díaz et al., 2021 (9)	3	1	2	6	Moderate
Lu et al., 2024	4	1	3	8	High
Poon & Leung, 2016	3	1	3	7	High
Rusaka & Rancāns, 2014 (22)	3	1	2	6	Moderate
Rutigliano et al., 2018 (26)	4	2	3	9	High
Yang et al., 2010 (30)	3	1	2	6	Moderate
Yu, 2011 (27)	2	1	2	5	Moderate
Liu, 2015 (28)	3	1	2	6	Moderate
Wu et al., 2020 (29)	3	1	2	6	Moderate

### Meta-analysis results

Relapse and transition to schizophrenia spectrum disorders were analyzed as separate outcomes. Transition to schizophrenia spectrum disorders referred to diagnostic reclassification from ATPD or brief psychotic disorder at baseline to schizophrenia, schizophreniform disorder, schizoaffective disorder, or related schizophrenia spectrum diagnoses during follow-up. Relapse referred to recurrence of psychotic symptoms, rehospitalization, or a new psychotic episode according to the definitions used in the original studies. These outcomes were not combined because relapse does not necessarily imply a change in diagnosis, and diagnostic transition can occur with or without relapse being explicitly reported.

### Transition to schizophrenia spectrum disorders

**Figure 2:**
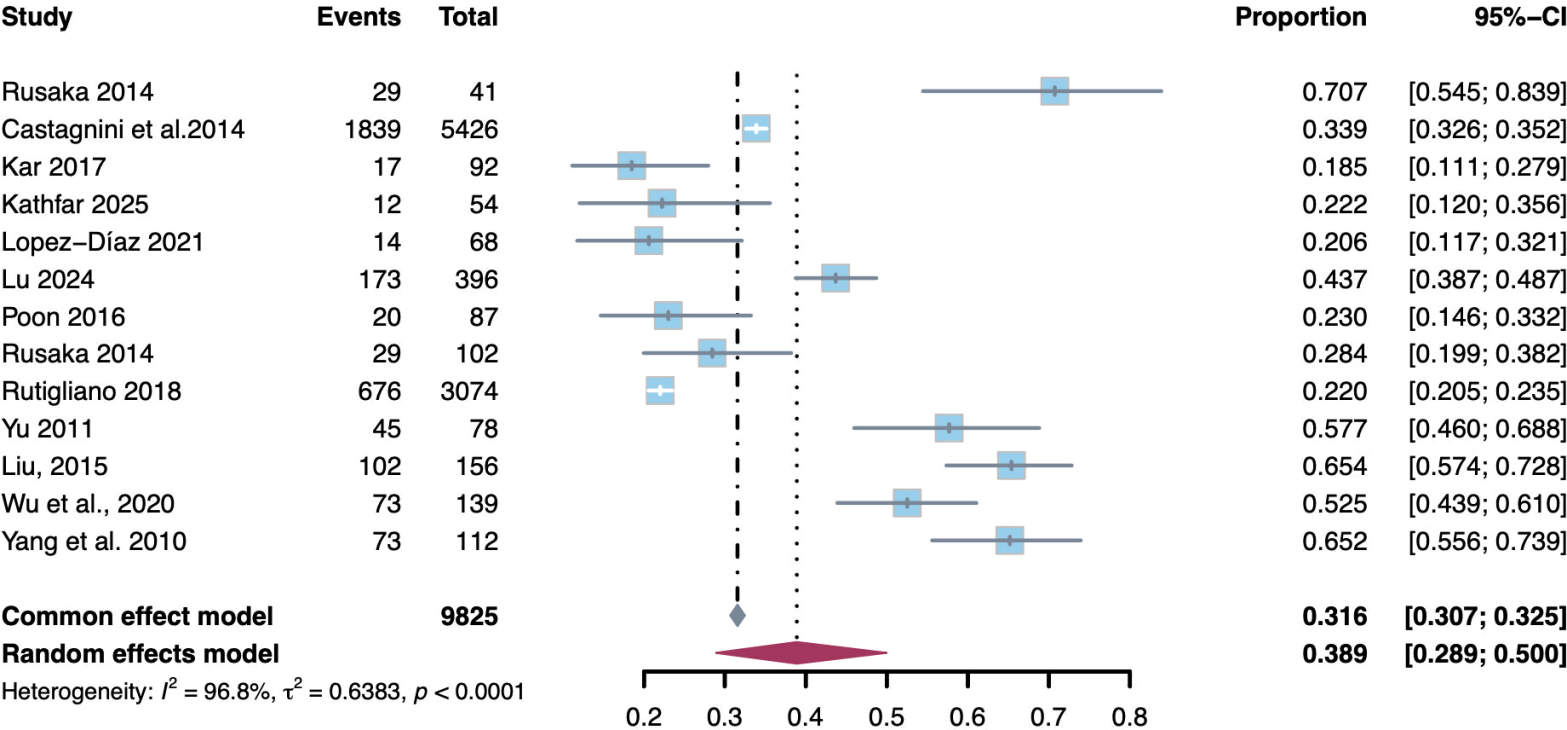
Forest plot of the proportion of transition to schizophrenia spectrum disorders in patients with ATPD.

Transition to schizophrenia spectrum disorders was examined in 14 studies. Considerable between-study heterogeneity was observed (I² = 96.8%, P < 0.001). The pooled proportion of transition to schizophrenia spectrum disorders was 0.39 (95% CI: 0.29–0.50) (**Figure 2**). Leave-one-out sensitivity analysis (**Supplementary Figure 1**) did not show that the pooled estimate was driven by any single study.

### Diagnostic stability

**Figure 3:**
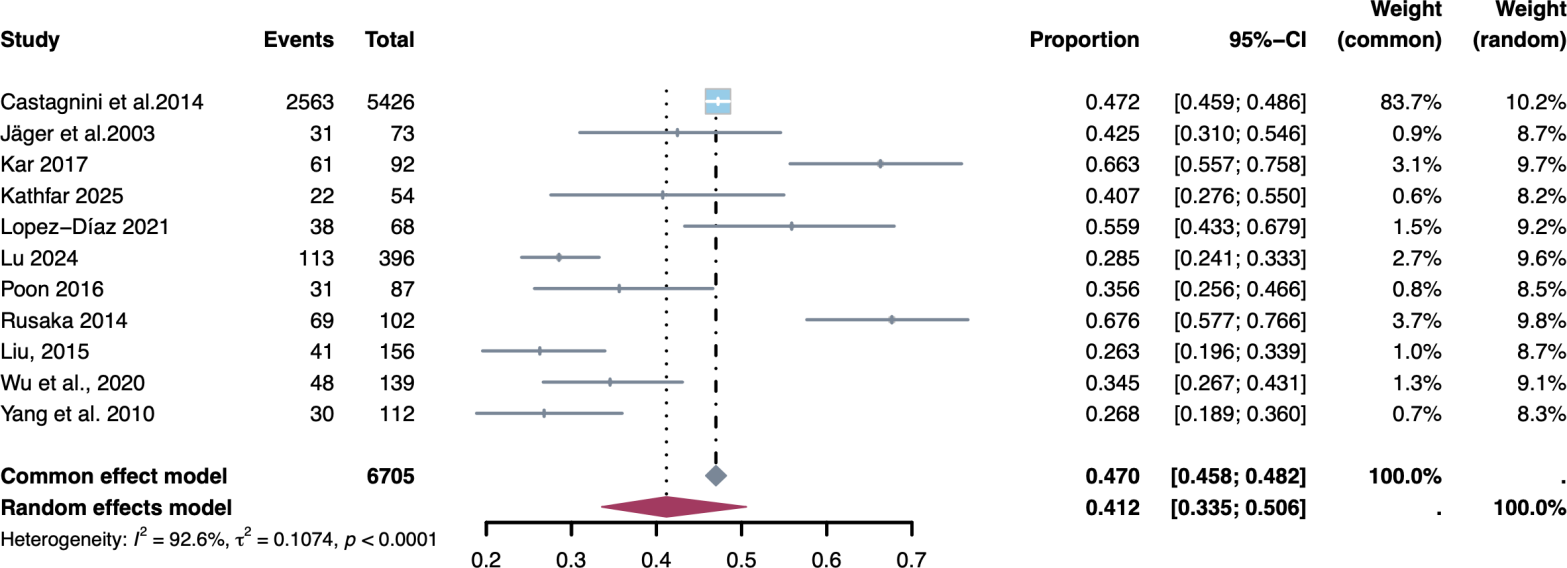
Forest plot of diagnostic stability in patients with ATPD.

The diagnostic stability of ATPD was examined in 11 studies. Considerable between-study variability was observed (I² = 92.6%, P < 0.001). Despite this heterogeneity, the pooled estimate derived from the random-effects model suggested that the proportion of patients retaining an ATPD diagnosis at follow-up was 0.41 (95% CI: 0.34–0.51) (**Figure 3**). Sequential omission of individual studies (**Supplementary Figure 2**) did not substantially affect the pooled estimate, suggesting stable results.

### Any diagnostic change

**Figure 4:**
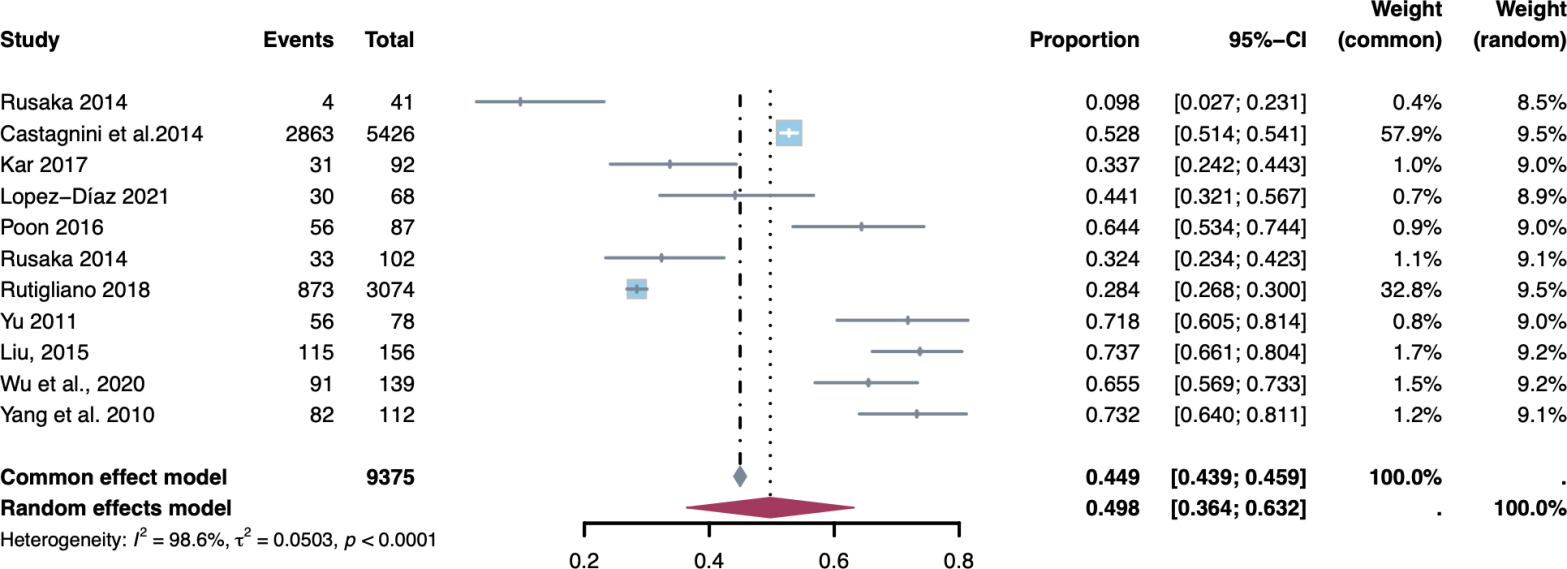
Forest plot of the proportion of any diagnostic change following an initial diagnosis of ATPD.

The occurrence of any diagnostic change following an initial diagnosis of ATPD was examined in 11 studies. Considerable between-study variability was observed (I² = 98.6%, P < 0.001). Despite this heterogeneity, the pooled estimate derived from the random-effects model suggested that the proportion of patients experiencing any diagnostic change was 0.50 (95% CI: 0.36–0.63) (**Figure 4**). Sequential omission of individual studies (**Supplementary Figure 3**) did not substantially affect the pooled estimate, suggesting stable results.

### Relapse

**Figure 5:**
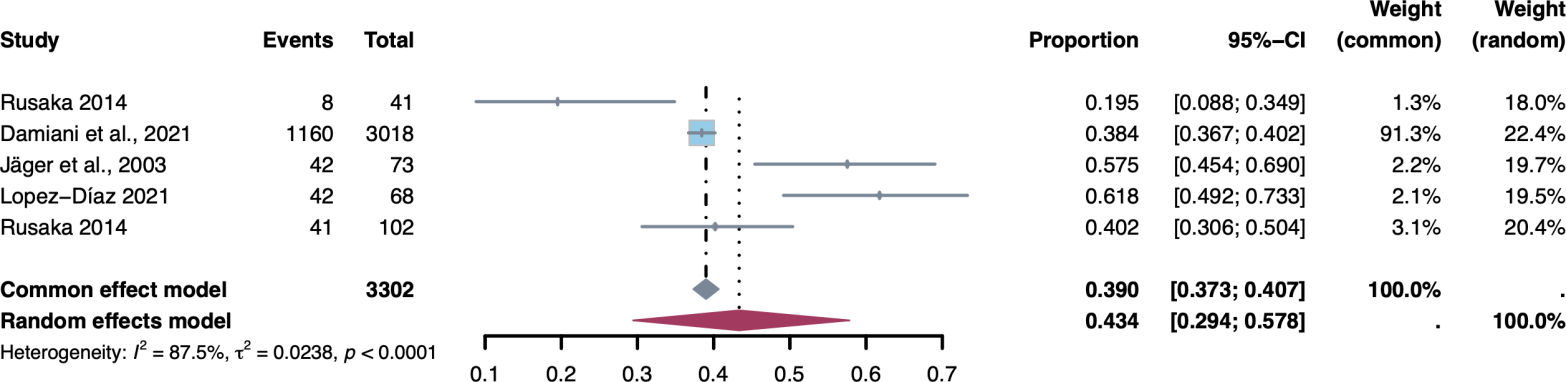
Forest plot of relapse in patients with ATPD.

Relapse was examined separately in 5 studies. Relapse definitions varied across studies and included recurrence of psychotic symptoms, rehospitalization, or a new psychotic episode. Considerable heterogeneity was observed (I² = 87.5%, P < 0.001). The pooled relapse proportion was 0.43 (95% CI: 0.29–0.58) (**Figure 5**). Because relapse and transition to schizophrenia spectrum disorders were reported by different subsets of studies and were based on different definitions, these pooled estimates should not be directly compared as mutually exclusive outcomes. Sequential omission of individual studies (**Supplementary Figure 4**) did not substantially affect the pooled estimate, suggesting stable results.

### Transition to mood disorders

**Figure 6:**
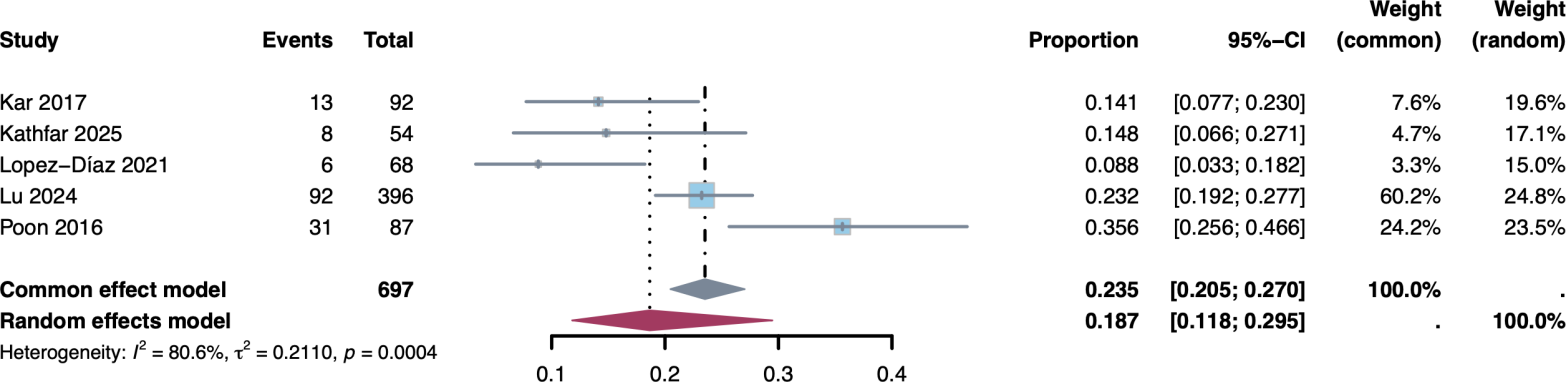
Forest plot of the proportion of transition to mood disorders in patients with ATPD.

The occurrence of transition to mood disorders following an initial diagnosis of ATPD was examined in 5 studies. Considerable between-study variability was observed (I² = 80.6%, P = 0.0004). Despite this heterogeneity, the pooled estimate derived from the random-effects model suggested that the proportion of transition to mood disorders was 0.19 (95% CI: 0.12–0.30) (**Figure 6**). Sequential omission of individual studies (**Supplementary Figure 5**) did not substantially affect the pooled estimate, suggesting stable results.

### Meta regression results

Exploratory univariable meta-regression analyses were conducted to assess whether selected study-level characteristics explained between-study heterogeneity (**Table 3**). The examined covariates included first-episode-only sample, publication year, country/region, study design, and diagnostic criteria. Publication year was coded as a continuous numeric variable, whereas the other covariates were coded as categorical factor variables. None of the examined covariates was significantly associated with transition to schizophrenia spectrum disorders, diagnostic stability, any diagnostic change, relapse, or transition to mood disorders. A non-significant trend was observed for first episode-only samples in relation to any diagnostic change (β = −0.27, P = 0.09). Overall, the substantial heterogeneity observed across outcomes was not explained by the available study-level covariates.

**Table 3:** Meta-regression analysis of potential sources of heterogeneity.

Covariate	Transition to schizophrenia spectrum disorders (β, P value)	Diagnostic stability (β, P value)	Any diagnostic change (β, P value)	Relapse (β, P value)	Transition to mood disorders (β, P value)
First episode only	0.15, 0.72	0.19, 0.45	-0.27, 0.09	0.29, 0.51	-0.51, 0.57
Publication year	-0.96, 0.15	0.12, 0.75	-0.33, 0.26	0.16, 0.46	0.17, 0.25
Country	-0.61, 0.67	0.11, 0.34	0.73, 0.86	-0.34, 0.29	-0.43, 0.91
Study design	0.16, 0.86	-0.77, 0.53	-0.11, 0.17	-0.26, 0.18	0.08, 0.39
Diagnostic criteria	-0.81, 0.16	-0.24, 0.75	-0.34, 0.77	0.38, 0.14	-0.19, 0.19

### Publication bias

Publication bias was assessed using funnel plots and Egger’s regression test (**Supplementary Figures S6**-**S10**). The results suggested a low likelihood of publication bias for transition to schizophrenia spectrum disorders (Egger P = 0.265), diagnostic stability (P = 0.395), any diagnostic change (P = 0.679), relapse (P = 0.561), and transition to mood disorders (P = 0.254). However, given the limited number of studies available for relapse and transition to mood disorders, these findings should be interpreted with caution.

## Discussion

### Summary of findings

In this systematic review and meta-analysis, we synthesized longitudinal evidence on the course of ATPD. Across studies, diagnostic stability appeared limited, with fewer than half of patients retaining the initial diagnosis over time. A substantial proportion of individuals experienced diagnostic change, and transitions to schizophrenia spectrum disorders were not uncommon, whereas transitions to mood disorders were observed less frequently. Relapse was reported in a notable subset of cases. Taken together, these findings suggest that ATPD may be characterized by considerable variability in its longitudinal trajectory, although the extent to which this reflects true clinical evolution, diagnostic uncertainty, or methodological heterogeneity remains unclear. Grover reviewed (31) a large body of Indian research on ATPD, including studies addressing course and outcome. However, their review had a broader narrative scope, whereas the present meta-analysis required extractable follow-up data aligned with predefined outcomes and avoided overlapping or non-ATPD-specific samples. Thus, the smaller number of included studies reflects the stricter eligibility criteria required for quantitative synthesis rather than a limited search alone. Nevertheless, the modest number of eligible studies limits the precision of some pooled estimates and reinforces the need for cautious interpretation.

### Interpretation of findings

The observed level of diagnostic instability raises questions regarding the boundaries and clinical utility of ATPD as a distinct diagnostic construct. While ATPD has traditionally been conceptualized as a brief and self-limiting condition, the present findings do not consistently support this assumption (32). Instead, ATPD may, in at least a subset of cases, represent an early or transitional presentation within a broader spectrum of psychotic disorders (6). However, it is important to acknowledge that diagnostic change over time may reflect not only underlying disease progression but also limitations in early diagnostic classification, particularly in the context of evolving symptom profiles (10).

The relatively high proportion of transitions to schizophrenia spectrum disorders may be interpreted within the framework of early psychosis, where initial presentations are often heterogeneous and diagnostically non-specific (33). In this context, ATPD diagnoses may capture a clinically diverse group of patients, some of whom later meet criteria for more persistent psychotic conditions (34). At the same time, differences in diagnostic systems, thresholds for reclassification, and clinical practice across settings may contribute to variability in reported transition rates. As such, the observed estimates should be interpreted with caution (35).

Transitions to mood disorders were less frequent, which may suggest a comparatively weaker association with affective trajectories. Nevertheless, the presence of such transitions indicates that ATPD cannot be viewed exclusively as a precursor to schizophrenia-spectrum conditions (31, 36). It remains possible that specific clinical phenotypes within ATPD, such as those with prominent affective symptoms, may follow distinct pathways, although the current evidence does not allow for firm conclusions in this regard (33).

Relapse was observed in a considerable proportion of patients, although the interpretation of this finding is complicated by variability in definitions across studies. In some cases, relapse was operationalized as rehospitalization, whereas in others it was based on broader clinical criteria (37, 38). This heterogeneity, combined with differences in follow-up duration, limits the comparability of relapse estimates and underscores the need for more standardized outcome measures.

### Methodological considerations and heterogeneity

A consistent feature of the present analysis was the high degree of between-study heterogeneity across all outcomes. I² values exceeded 80% in most analyses and approached extreme levels in some cases. Although meta-regression was conducted, the examined study-level variables did not account for this variability. This suggests that unmeasured factors—such as differences in follow-up intensity, treatment exposure, case ascertainment, and diagnostic practices—may play a substantial role. Variability in follow-up duration is likely to have influenced the observed estimates, as longer observation periods increase the likelihood of detecting both diagnostic transitions and relapse. Additionally, differences between ICD-based and DSM-based frameworks, as well as potential inconsistencies in applying diagnostic criteria, may further contribute to heterogeneity. These considerations limit the interpretability of pooled estimates and highlight the importance of cautious inference. The high heterogeneity observed across all primary outcomes is a major limitation of this review and should be considered when interpreting the pooled estimates. Although random-effects models and leave-one-out sensitivity analyses were applied, these approaches cannot fully eliminate the influence of clinical and methodological diversity across studies. Differences in diagnostic systems, follow-up duration, case ascertainment, treatment exposure, relapse definitions, and thresholds for diagnostic reclassification may all have contributed to variability in the results. Therefore, the pooled proportions should not be interpreted as precise prognostic probabilities for individual patients, but rather as approximate summary estimates reflecting the currently available longitudinal evidence.

Relapse and diagnostic transition should be interpreted as related but distinct longitudinal outcomes. Relapse reflects recurrence of psychotic symptoms, rehospitalization, or a new psychotic episode, depending on the definition used in the original study. In contrast, transition to schizophrenia spectrum disorders reflects diagnostic reclassification over time. A patient may relapse without receiving a new diagnosis, and diagnostic conversion may be recorded even when relapse is not separately reported. Therefore, the pooled relapse estimate should not be directly compared with the pooled estimate for transition to schizophrenia spectrum disorders as if they were mutually exclusive outcomes.

Exploratory meta-regression analyses did not identify first-episode-only sample, publication year, country/region, study design, or diagnostic criteria as statistically significant sources of heterogeneity. However, these null findings should be interpreted cautiously because the analyses were based on study-level data, the number of included studies was limited, and several potentially important covariates, including age, sex distribution, and follow-up duration, were not consistently reported across studies. Thus, the analyses were likely underpowered and cannot exclude the possibility that demographic or follow-up characteristics influence the long-term course of ATPD.

### Comparison with clinical high-risk syndromes

The present findings may also be interpreted in relation to clinical high-risk (CHR) syndromes for psychosis, particularly brief limited intermittent psychotic symptoms (BLIPS). BLIPS and ATPD share some clinical features, including brief psychotic symptoms, rapid remission, and heterogeneous longitudinal outcomes. However, they are not equivalent constructs (39). BLIPS is generally conceptualized as a CHR risk syndrome and usually refers to brief, self-limiting psychotic symptoms that do not meet the duration threshold for a sustained psychotic disorder. In contrast, ATPD is an ICD diagnostic category applied after an acute psychotic episode that meets criteria for a psychotic disorder and is expected to remit within a short period (40). Both CHR syndromes and ATPD may be followed by remission, persistence of symptoms, relapse, or later transition to schizophrenia spectrum or affective disorders. However, transition estimates should not be directly compared across these populations because they differ in diagnostic thresholds, symptom duration, ascertainment context, treatment exposure, and classification systems. This overlap highlights the broader diagnostic fluidity of early psychosis while also supporting the need for ATPD-specific longitudinal evidence. Sample age may also influence the long-term course of ATPD. Younger patients with acute psychotic presentations may be more closely aligned with early-psychosis trajectories, in which diagnostic boundaries are often unstable and later transition to schizophrenia spectrum disorders may emerge over time. Later-onset brief psychotic episodes may reflect different clinical contexts, including affective, medical, substance-related, or stress-associated factors. However, age was inconsistently reported across the included studies, and age-stratified outcome data were rarely available. Therefore, we could not formally assess whether age modified diagnostic stability, relapse, or transition risk. Future studies should report age-specific outcomes and examine age at onset as a potential predictor of ATPD course.

### Clinical implications

The present findings do not lend themselves to straightforward clinical conclusions. While the data suggest that ATPD may not be uniformly stable over time, the substantial heterogeneity and variability across studies make it difficult to draw definitive implications for individual patient care. Rather than supporting a specific clinical pathway, these findings may be better understood as reflecting the inherent uncertainty associated with early psychotic presentations. In this context, ATPD may function less as a stable diagnostic category and more as a provisional label, the meaning of which evolves over time. This perspective may have implications for how clinicians conceptualize early psychotic disorders, although further evidence is needed before firm recommendations can be made.

### Strengths and limitations

This study has several strengths, including the comprehensive synthesis of longitudinal data across diverse populations and settings, and the application of systematic meta-analytic methods. The inclusion of studies from both Western and Asian contexts may enhance the breadth of the evidence base.

However, several limitations should be considered. The high level of heterogeneity represents a major challenge and limits the confidence that can be placed in pooled estimates. Differences in diagnostic criteria, outcome definitions, and follow-up duration further complicate interpretation. In addition, the relatively small number of studies available for certain outcomes, such as relapse and transition to mood disorders, may reduce the precision of these estimates. Finally, as all included studies were observational, the findings are subject to potential bias and confounding.

### Future directions

Future research may benefit from more standardized approaches to defining and measuring outcomes in ATPD. Prospective studies with clearly defined diagnostic criteria and consistent follow-up protocols may help clarify the extent to which ATPD represents a distinct clinical entity versus a transitional state. In addition, greater attention to potential predictors of outcome—such as symptom profiles, biological markers, and treatment variables—may help to identify subgroups with differing trajectories. Such work may contribute to a more nuanced understanding of ATPD and its place within the broader spectrum of psychotic disorders.

## Conclusion

The available longitudinal evidence suggests that ATPD has limited diagnostic stability in a substantial proportion of patients, with diagnostic transition—particularly to schizophrenia spectrum disorders—reported in many cohorts. However, the high degree of between-study heterogeneity, differences in follow-up duration and outcome definitions, and the observational nature of the included evidence limit the certainty of these estimates. Accordingly, the findings should be interpreted cautiously and should not be viewed as definitive prognostic probabilities. Further prospective studies using standardized diagnostic criteria, follow-up protocols, and outcome definitions are needed to better clarify the long-term clinical trajectory of ATPD.

## Data Availability

The original contributions presented in the study are included in the article/**Supplementary Material**. Further inquiries can be directed to the corresponding authors.
