# Supplementary material for: Diagnostic stability and long-term outcomes of acute and transient psychotic disorders: a systematic review and meta-analysis
Source: Front Psychiatry. 2026 Jul 10;17:1839599. doi: 10.3389/fpsyt.2026.1839599 (PMC13398203; doi:10.3389/fpsyt.2026.1839599)
Supplement: Supplementary file 1 [file DataSheet1.docx]

Table S1 search strategy

(“acute and transient psychotic disorder” OR “acute transient psychotic disorder” OR ATPD OR “brief psychotic disorder” OR “brief reactive psychosis” OR “reactive psychosis” OR “short-lived psychosis” OR “first episode psychosis” OR “first-episode psychosis” OR “first admission psychosis” OR “first-admission psychosis” OR “early psychosis”) AND (“diagnostic stability” OR “diagnostic transition” OR “diagnostic change” OR conversion OR relapse OR outcome OR course OR prognosis OR follow-up)


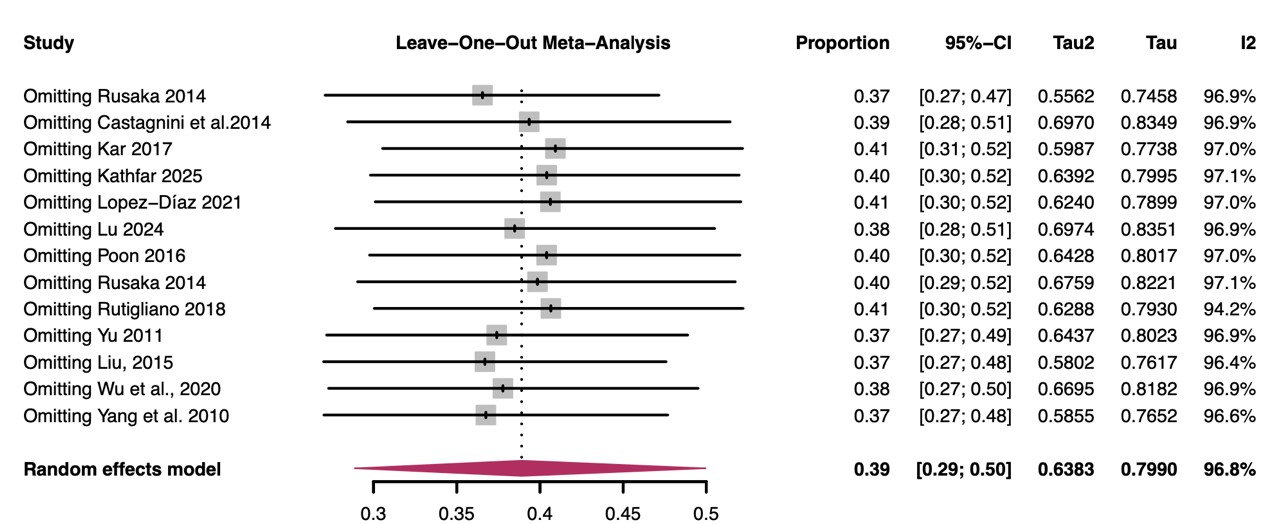


Figure S1 Sensitivity Analysis of schizophrenia spectrum disorders


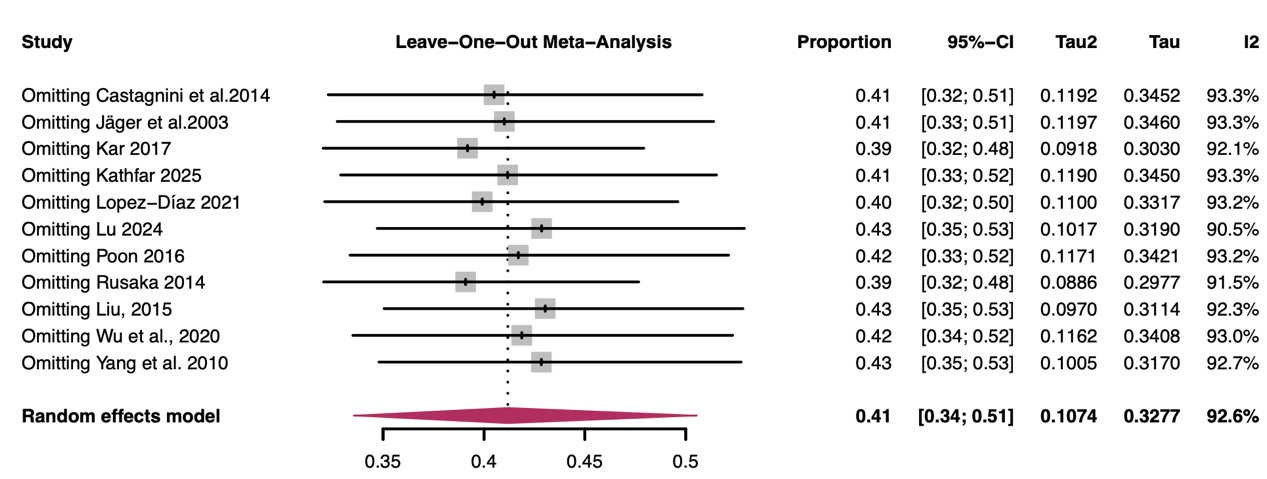


Figure S2 Sensitivity Analysis of diagnostic stability


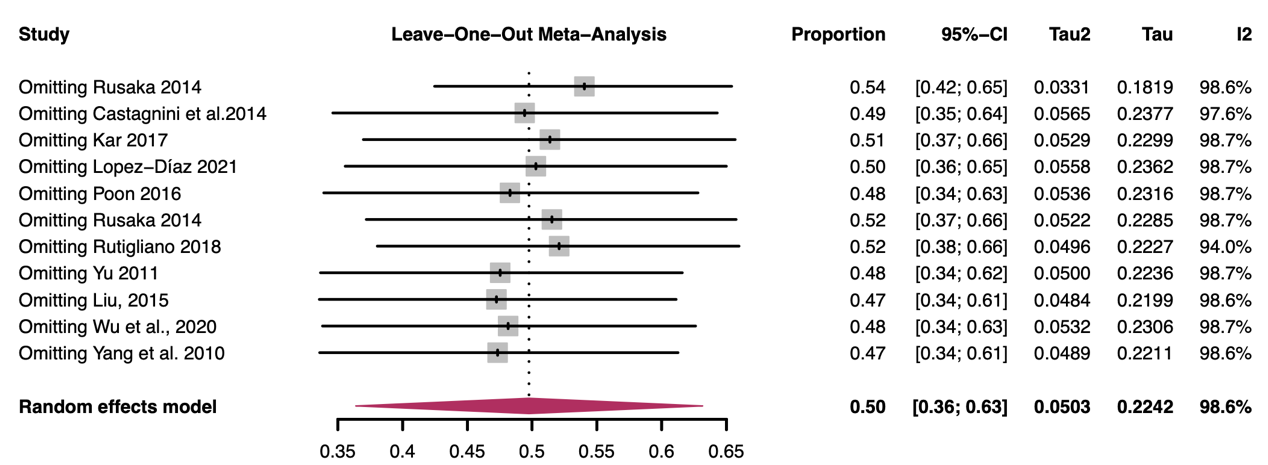


Figure S3 Sensitivity Analysis of any diagnostic change


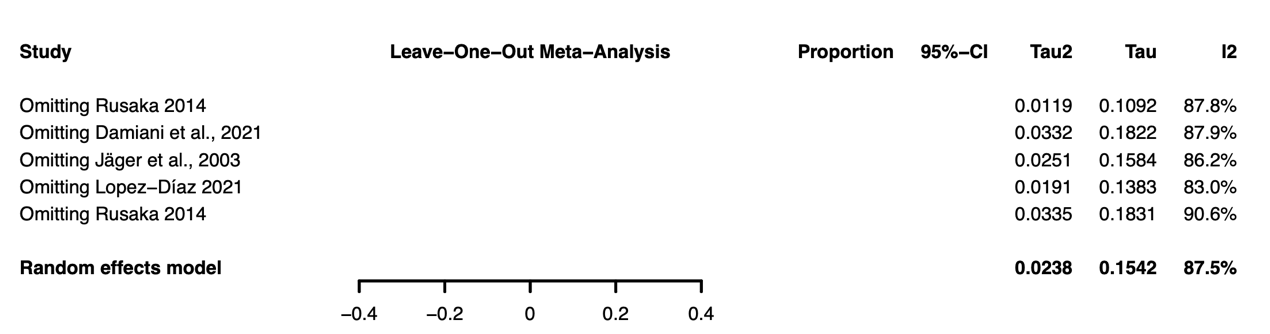


Figure S4 Sensitivity Analysis of relapse


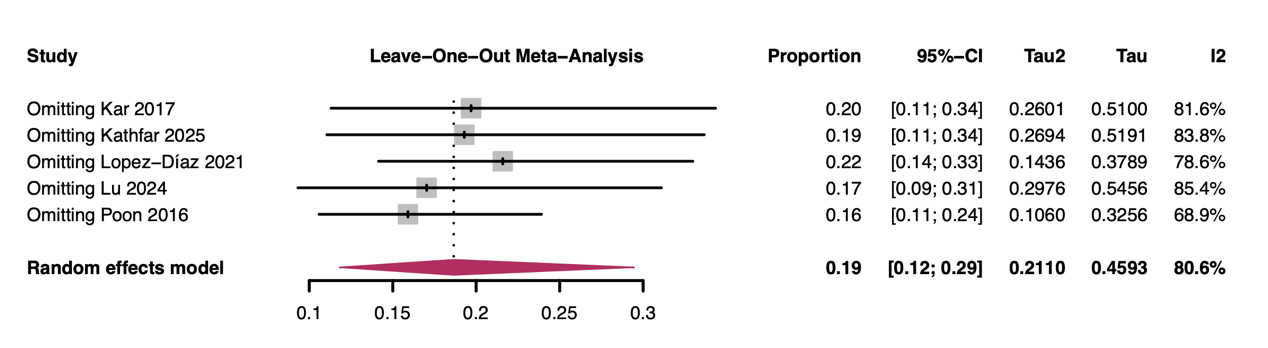


Figure S5 Sensitivity Analysis of transition to mood disorders

Figure S6 Funnel plot of the meta-analysis of transition to schizophrenia spectrum disorders

Figure S7 Funnel plot of the meta-analysis of diagnostic stability

Figure S8 Funnel plot of the meta-analysis of any diagnostic change

Figure S9 Funnel plot of the meta-analysis of relapse

Figure S10 Funnel plot of the meta-analysis of transition to mood disorders
